# Can Positive Affective Variables Mediate Intervention Effects on Physical Activity? A Systematic Review and Meta-Analysis

**DOI:** 10.3389/fpsyg.2020.587757

**Published:** 2020-11-05

**Authors:** Cheng Chen, Emily Finne, Alexandra Kopp, Darko Jekauc

**Affiliations:** ^1^Institute of Sports and Sports Science, Karlsruhe Institute of Technology, Karlsruhe, Germany; ^2^Department Prevention and Health Promotion, School of Public Health, Bielefeld, Germany; ^3^Department of Sport Science, Institute of Sport Sciences, Humboldt University of Berlin, Berlin, Germany

**Keywords:** intervention, positive affective variable, physical activity, mediation, meta-analysis

## Abstract

Well-developed theories are necessary to guide the public in increasing physical activity (PA) and promoting physical health. The role of positive affective variables (PAVs) in exercise is gaining more attention, but none of the literature has provided a systematic review and quantitative analysis of its mediating role. Therefore, the purposes of this study are (1) to systematically review studies of PA interventions, that use PAVs as the mediating variables, in order to evaluate and provide narrative summaries of these studies; (2) to statistically synthesize evidence for the mechanism of the effects of PAVs on PA outcomes. To conduct an extensive search, a PRISMA-compliant protocol was completed, and five electronic databases had been searched by 1 April 2020. We used a two-stage structural equation modeling (TSSEM) analysis approach to test how interventions trigger the critical PA change process to influence outcomes. The search strategy generated 1,732 papers potentially relevant to this study; forty of these studies met the data extraction criteria for meta-analytic mediation analysis. The path coefficient from intervention to PAV *a* = 0.26 (95% CI = 0.08 to 0.44), the path coefficient from PAV to PA *b* = 0.21 (95% CI = 0.13 to 0.28), and the direct effect from intervention to PA is also significant (*c* = 0.19, 95% CI = 0.12 to 0.26). In addition, the indirect effect of intervention on PA via PAV was statistically significant (*c*' = 0.05, 95% CI = 0.02 to 0.10). This reveals that PAVs partially mediate the relationship between interventions and PA. Our study is the first to systematically summarize the effects of experimental studies to increase PA through PAVs. It is highly recommended to make future interventions more innovative and to target the PAVs as mediators with higher fidelity.

## Introduction

A growing body of empirical research shows that regular physical activity (PA) is effective in improving a range of clinical and non-clinical health-related outcomes, including metabolic disorders (Denham et al., 2016), cardiorespiratory fitness (Shuval et al., 2014), arterial stiffness (Boreham et al., 2004) and physical and psychological well-being (Penedo and Dahn, 2005). Indeed, though PA is so fundamental to human's health, a minority of adults report engaging in PA at a level compatible with public health guidelines, countering the 50% of people who stop exercising within the first 6 months of starting an exercise program (Finne et al., 2019). Physical activity maintenance has proven to be a daunting and enduring challenge for PA and public health professionals, as the benefits of PA depend entirely on constant engagement (Annesi, 2003). Therefore, the psychological mechanism that underlies PA persistence has come into sharp focus.

To date, the dominant theoretical approaches employed to intervene in PA include the social cognitive theory (Bandura, 1998), the theory of planned behavior (Ajzen, 1991), and the transtheoretical model (Prochaska and Velicer, 1997). However, even as the most predictable framework, social cognitive theory, on average, can only explain 20% of the variance in PA maintenance (Jekauc et al., 2015). The dominance of these theories hinders the development of theories, because they focus merely on cognitive mechanisms and neglect the role of affective variables (Jekauc and Brand, 2017). Thus, an extension of the theories for affective variables seems inevitable (Jekauc et al., 2015). Considering that many exercisers are susceptible to negative affects during PA procedures (Ekkekakis and Acevedo, 2006; Rose and Parfitt, 2010), an emphasis on positive affects may have a positive impact on adherence to exercise with inevitable motivational effects. Somewhat also related to this notion, Parfitt and Hughes (2009) elucidated the implications of the peak-end rule, which states that the affective experience of an exerciser can have a potent effect in guiding future participation decisions (Williams et al., 2012) via the proposed mechanism of affective memory (Fredrickson and Kahneman, 1993).

Primarily, the words emotional or affective apply, to varying degrees, to an ill-defined, board, and heterogeneous aggregate of phenomena (Fehr and Russell, 1984). Today, we consider the term of affect concerning a neurophysiological state that is consciously accessible as a pure primitive non-reflective feeling (Russell and Barrett, 1999). In contrast, emotion refers to feelings that are typically brief, intense, and attributable to an apparent cause (Beedie et al., 2005). Rather than thinking about emotional feelings in terms of categories, an alternative way to organize them is to arrange them along dimensions. Emotions can be conceptualized in the form of several dimensions, and these dimensions can be independent of each other, such as positive and negative activation (Watson et al., 1999), or positivity and negativity (Cacioppo and Gardner, 1999). According to existing research, the proximity-avoidance distinction is applicable in emotions (positive and negative affective dispositions) (Watson et al., 1999), and the neurological basis for this distinction between motivation and emotion has been demonstrated through affective neuroscience (Davidson, 2003). As stated by Larsen et al. (2008), “motivation and valence tend to be correlated, such that positive emotions are associated with approach and negative emotions with avoidance.” For these reasons, we will concentrate on affective variables, not on negative affective variables. In other words, this paper will generalize non-negative, positive affects, emotions, and feelings, and will use the term positive affective variables (PAVs) to refer to them.

The effects of PAVs have been subject to investigation in behavior change contexts, resulting in several theoretical and empirical studies. According to van Cappellen et al. (2018), the upward spiral theory of lifestyle change states that positive affect experienced during health behaviors increases incentive salience for cues associated with those behaviors, which in turn, implicitly guides attentions and the everyday decisions to repeat those behaviors. Fredrickson's broaden-and-build theory argues that positive affect builds a suite of endogenous resources, which may, in turn, amplify the positive affect experienced during positive health behaviors and strengthen the non-conscious motives. Similarly, consistent with hedonic theories of behavior (Cabanac, 1992), where persistent behaviors are considered to be determined by positive reinforcement, core affective valence in response to PA has been posited as an essential determinant of future PA behavior (Bryan et al., 2007; Williams, 2008). Empirical studies also supported this idea; for example, Klusmann et al. (2015) found that the fulfillment of emotional outcome expectancies emerges as a significant predictor of adoption and maintenance of PA. Similarly, Schutte et al. (2017) found that positive affective responses were associated with higher amounts of regular exercise activity and that this association was accounted for by an overlap in genetic factors influencing affective responding and exercise behavior.

In contrast to the broad evidence base for PAVs' effectiveness, relatively few studies have tested the mechanisms of PAVs in exercise interventions. Mediators have been defined as intervening variables in the causal process or pathway between intervention and PA (Diener and Emmons, 1984). Given its propensity to optimize intervention effects through identifying potential psychological mechanisms underlying PA intervention, matching exercise intervention prescription to the theoretical framework, and strengthening active components of interventions during PA seems reasonable. It is a worthy venture to investigate PAV as a mediator of PA outcome (Kazdin, 2007).

So far, three reviews have summarized the classification of mediators of PA (Lewis et al., 2002; Rhodes and Pfaeffli, 2010; Murray et al., 2018); however, research into the mediation role of PAV have been narrow, incomplete, and problematic, due to the somewhat limited sample size. For instance, Lewis et al. (2002) examined three studies that investigated enjoyment as a mediator of intervention and PA and indicated that two of them were not significant. Murray et al. (2018) integrated findings with experimental data to propose that the mechanism through emotion works, and wherein half of the empirical studies reported significant findings. Nonetheless, Klos et al. (2020) and Rhodes and Pfaeffli (2010) showed moderate evidence of interventions in increasing enjoyment and PA. In contrast, the mediating effect of PAV in exercise interventions remain to be examined. Therefore, the purpose of this study is two-fold. First, it aims to systematically review studies of PA interventions that use PAV as the mediating variable to evaluate and provide general summaries (study, participant, measurement, and intervention characteristics) of these studies. Of which, study and participant characteristics include research setting, PA level at baseline, percentage of female subjects, sample size, and mean age; measurement characteristics include types and methods of PA and PAV measurement; intervention characteristics include theory, length of intervention, and behavior change techniques used in each study). Second, it aims to statistically synthesize evidence for the mechanism of the effect of PAV on PA outcome. The combination of statistical synthesis and narrative summaries of existing mediation findings will allow us to draw more reliable and comprehensive conclusions about how PAVs improve PA, compared to using either one of these techniques in isolation (Gu et al., 2015).

## Methods

### Search Strategy

**Figure 1:**
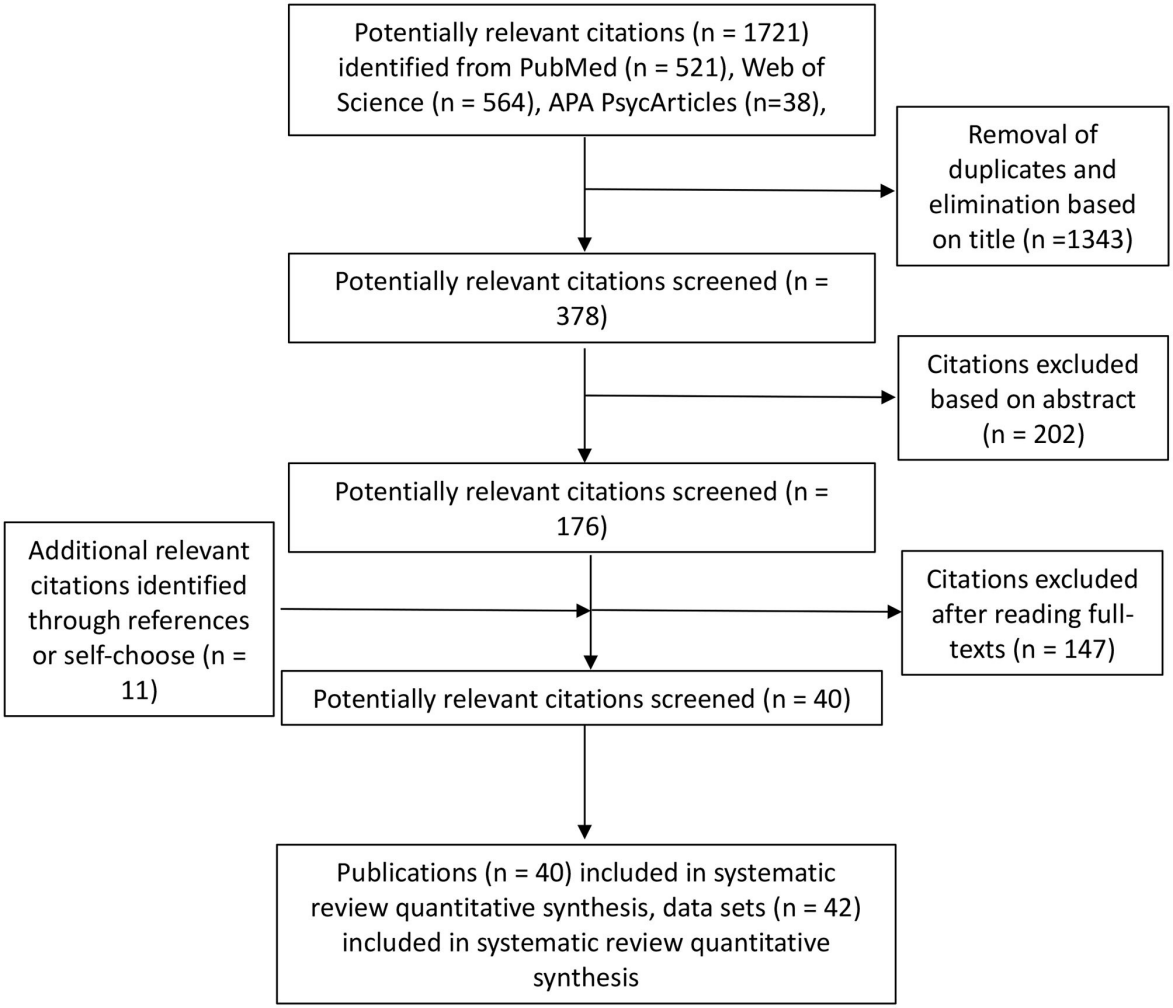
PRISMA flow diagram for articles identified, screened eligible, and included in this paper.

A protocol using the PRISMA standards (Moher et al., 2009) was completed before initiating the literature search (Figure 1). A comprehensive search of published studies up to 01/04/2020 was conducted using the following electronic databases: Web of Science, PubMed, PsycINFO, PsycArticle, and Psychology and Behavioral Sciences Collection. The search term was: (1) Intervention OR Trial OR Experiment; (2) Physical Activity OR Exercise; (3) Enjoy^*^ OR Affect^*^ OR Emotion^*^ OR Mood^*^ OR Feeling; (4) Mechanism^*^ OR Mediat^*^ OR Predict^*^ OR Process^*^ OR “Structural equation modeling” OR Caus^*^ OR Path^*^ OR Correlat^*^ OR Relationship OR Associat^*^; (5) NOT (Patient^*^ OR Cancer OR clinical OR disease^*^ OR Illness OR Depression OR Rat OR Mouse OR Protocol OR Cell OR Bone^*^ OR Blood OR Rehabilitation OR Disorder^*^ Injur^*^ OR HIV OR Carbohydrate OR Athlete^*^ OR Player^*^ OR Runner^*^ OR Review OR Comment OR Therapy); (6) 1 AND 2 AND 3 AND 4 AND 5.

For inclusion, each study was required to meet the following criteria: (1) intervention studies that assessed the PAV as a putative mediator of PA; (2) studies' objectives were to increase lifestyle or recreational PA through affective variables not for competitive sports or fitness; (3) information needed to calculate effect sizes must have been made available for PAVs and PA (PA measurement could be self-reported or objective measured, e.g., accelerometer readings); (4) participants are from a healthy population (non-clinically defined populations, obese or pregnant populations were also excluded); (5) written in English;(6) original, peer-reviewed studies. Furthermore, similar dimensions (e.g., positive affect, PA enjoyment, PE enjoyment, revitalization, positive engagement, and remembered pleasure) were identified as PAVs, and negative affective variables were excluded. We intentionally selected the shortest duration of 10 min for PA, given that 10 min is the shortest recommended duration of exercise to elicit health benefits (Edwards and Loprinzi, 2019).

To evaluate mediators between intervention and PA, an additional criterion was established based on Murray et al. (2018). An included study had to involve at least one of the following: “(a) formal mediation tests, (b) examined association of putative mediators (or mediator changes) with PA outcomes (or PA changes), (c) examined intervention effects on putative mediators.”

### Data Extraction and Data Analysis

Searches were completed and the eligibility of each study was determined by the first author. Abstracts were cross-checked against the inclusion criteria. Where the first author was unsure of relevance, the abstract was retained, and decisions regarding inclusion and exclusion were resolved by discussion with the last author. A study that can fulfill the data extraction criteria below is eligible for our meta-analysis.

According to Stone et al. (2019), stratification by quality in meta-analysis leads to a form of selection bias (collider stratification bias), and it is recommended for inclusion in all eligible studies rather than removing studies with low-quality ratings. Therefore, this paper does not evaluate and grade the studies' quality but includes all eligible studies.

To understand how change occurs during interventions, evaluating mediation effect is essential [i.e., how an intervention (X) influences an outcome (Y) through a mediator (M)] (Kazdin, 2007). Accordingly, we used a two-stage structural equation modeling (TSSEM) approach to test how interventions trigger the critical PA change process to influence outcomes (Cheung, 2014). The metaSEM package in R was used to perform our analyses (Cheung, 2019). In the first stage, we combined the relative effect sizes into matrices to calculate a pooled correlation matrix; the second stage involved treating the pooled matrix as the observed correlation matrix and fitting a structural mediational model to the matrix to test the fit of the model to the data. The specification of any structural model in the metaSEM package is done by using two matrices, of which matrix A specifies all regression coefficients in the model, and matrix S specifies all variances and covariance in the model (McArdle and McDonald, 1984). The procedure used is as explained by Jak (2015).

In the preparation phase, the bivariate correlations between X (intervention vs. control/pre-intervention), intervention change in PAVs (M), and PA (Y) were extracted from each relevant study. If a study did not explicitly report bivariate correlation coefficients, we used t-statistics, *F*-statistics, means, standard deviations, and effect sizes to calculate bivariate correlations (Lipsey and Wilson, 2001). Studies in which only reporting regression coefficients were omitted from the mediation analysis, as results from both the existing meta-analysis and the Monte Carlo simulations revealed that beta estimation procedures were associated with potentially significant biases (Peterson and Brown, 2005; Roth et al., 2018). To ensure that the observations in the sample were independent (Hunter and Schmidt, 2004), only one PAV/PA outcome was selected from each study to enable bivariate correlations to be extracted. Although it would be possible to calculate mean correlations across multiple outcomes in a single study, it would not be straightforward to determine the appropriate sampling variance of averaged correlations. Besides, we collected descriptive data from the included studies, such as setting, subjects' PA level at baseline, percentage of female subjects, the theoretical basis of the intervention, and PAV and PA measurement types and methods. In particular, the PA level at baseline can be divided into four categories according to whether the subjects meet a PA guideline (which can be defined arbitrarily by each study): not meeting PA guideline at baseline, meeting PA level at baseline, mixed and unreported. In order to gain a clearer understanding of the studies' intervention methodologies, we extracted data for each study's behavior change techniques based on Michie et al. (2011) 40-item taxonomy. The coding of the behavior change techniques was also primarily done by the first author, but for those codings that could not be determined by the first author, decisions were discussed with the last author.

## Results

### Study Flow and Characteristics

The search strategy generated 1,732 papers potentially relevant to this study; we excluded 1,692 papers following the eligibility criteria (e.g., unrelated topics, chronic condition, qualitative studies, insufficient data). After initial exclusions, there were 176 articles for full-text review, of which 11 were identified by cross-referencing. Of the 40 included studies which fulfilled the data extraction criteria of meta-analytic mediation analysis (see Figure 1 and Appendix 3 in Supplementary Material), two included a measure of two independent subgroups (Digelidis et al., 2003; Hutchinson et al., 2018). Hence, a total of 42 data sets were elicited for analyses.

A summary of the data from the 40 articles included in this paper is presented in Appendix 1 in Supplementary Material. In terms of the participants' age, four age intervals were designed for distinguishing and classifying the mean age of each study; they are the interval of study mean age below 18 (*n* = 18), the interval of study mean age between 18 and 35 (*n* = 13), the interval of study mean age between 36 and 60 (*n* = 7), and the interval of study mean age over 60 (*n* = 2). In terms of gender distribution, just one study identified its gender as male and ten studies delimited their gender as female, the genders of the subjects in the remaining 28 studies were mixed. In terms of physical activity at baseline, we marked out four classifications as “not meeting PA guidelines at baseline” (*n* = 18), “meeting PA guideline at baseline” (*n* = 3), “mixed” (*n* = 9), and “unreported” (*n* = 10). Besides, the primary constructs of mediating variables (PAVs) measured in these studies were enjoyment (*n* = 25), affect (*n* = 5), affective attitude (*n* = 4), affective valence (*n* = 2), exercise-induced feeling (*n* = 1), remembered pleasure (*n* = 1), and mood state (*n* = 1). Thirty-six intervention studies explicitly mentioned theoretical underpinnings in their descriptions; the other four intervention studies did not mention any framework. The most commonly used theoretical frameworks were: the social cognitive theory (*n* = 12), the self-determination theory (*n* = 8), the transtheoretical model (*n* = 7), the theory of planned behavior (*n* = 7), and the dual-mode model (*n* = 6). Approximately 60% of the studies were conducted in schools or at universities (*n* = 24), the remaining study settings varied (such as in laboratories, communities, outdoors, workplaces, internet, homes, gyms).

The intervention techniques employed by each study are summarized in detail in Appendix 2 in Supplementary Material. According to Michie et al. (2011), the 40 studies used 2 to 17 behavior change techniques, of which five studies employed no more than three behavior change techniques, 27 studies employed 4 to 10 intervention techniques, and 18 studies employed more than ten behavior change techniques. In terms of the frequency of use of each behavior change technique, the most commonly used intervention techniques were (1) provide instruction on how to perform the behavior (*n* = 32), (2) action planning (*n* = 25); (3) Model/demonstrate the behavior (*n* = 24); (4) Plan social support/social change (*n* = 23); (5) Stress management/emotional training (*n* = 21). However, five other behavior change techniques were not employed by any of the included studies: (1) Prompt generalization of a target behavior; (2) Prompt identification as a role model/position advocate; (3) Prompt anticipated regret; (4) Fear arousal; (5) Stimulate anticipation of future rewards.

### The Mediating Role of Positive Affective Variables

We then report the results of the TSSEM analysis in a stepwise sequence. For calculating the pooled correlation matrix, we used the 42 correlation matrices. In a first step, we tested a fixed-effects model. The χ^2^ of the model with equality constraints on all correlation coefficients across studies was significant $\chi_{\left(45\right)}^{2}$ = 196.48, *p* < 0.01, CFI = 0.719, CLI = 0.701, and the RMSEA was larger than 0.10, indicating a bad suitability. Thus, the random-effects model seems more appropriate (Harrer et al., 2019). The total pooled sample size was 9,235. The averaged correlation matrix based on the random-effects model was shown in Table 1. According to Gignac and Szodorai (2016) suggested that in interpreting statistical results, correlations of 0.10, 0.20, and 0.30 should be considered relatively small, typical, and relatively large, and we found medium-sized overall correlations between intervention and PAV (*r* = 0.26, *p* < 0.01), PAVs and PA (*r* = 0.25, *p* < 0.001), and intervention and PA (*r* = 0.25, *p* < 0.001).

**Table 1:** Pooled correlation coefficients (*k* = 42) for X (participants in post-intervention vs. post-control/ pre-intervention), M (PAV) and Y (PA).

	**X**	**M**	**Y**
X	1		
M	0.26^**^	1	
Y	0.25^***^	0.26^***^	1

** *p < 0.01*,

*** *p < 0.001*.

**Figure 2:**
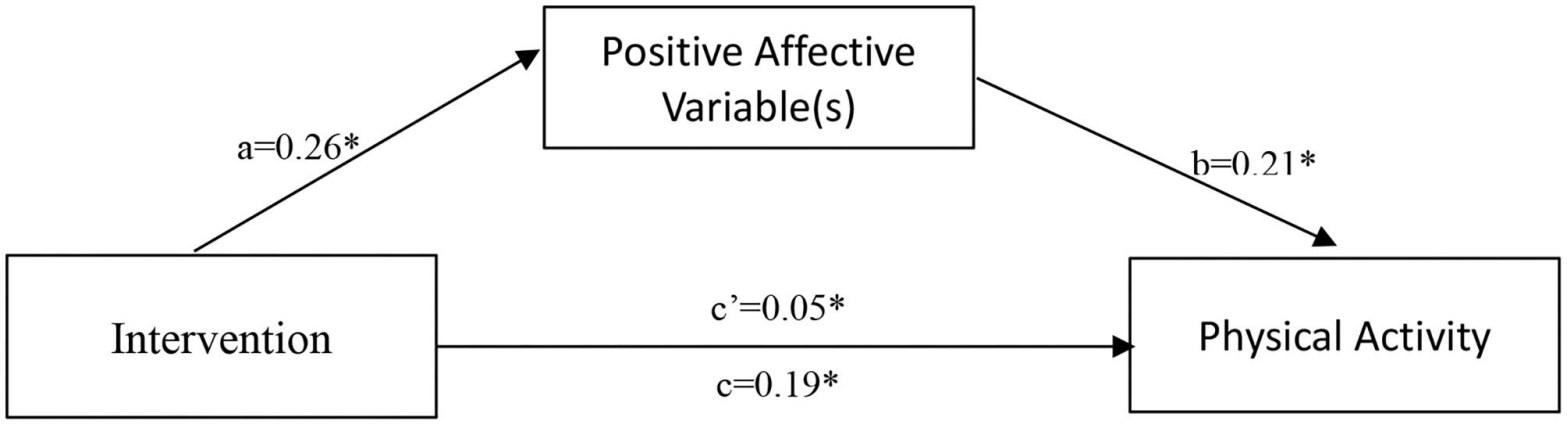
Path diagram depicting the mediational model for intervention to PA with PAV(s) as the mediator. Values are path coefficients. ^*^p < 0.05. a = “path coefficient from intervention to PAV,” b = “path coefficient from PAV to PA,” c = “direct effect from intervention to PA” and c' = “indirect effect from intervention to PA.”

In stage 2, we used the pooled correlation matrix to fit the hypothesized structural model. Figure 2 displayed the path diagram of the mediational model. The path coefficient from intervention to PAV *a* = 0.26 (95% CI = 0.08 to 0.44), the path coefficient from PAV to PA b = 0.21 (95% CI = 0.13 to 0.28), and the direct effect from intervention to PA is small but significant (*c* = 0.19, 95% CI = 0.12 to 0.26). In addition, the indirect effect of intervention on PA via PAV was small (*c*' = 0.05, 95% CI = 0.02 to 0.10). Since zero is not included in the 95% confidence interval, the indirect effect can be considered small but significant. This provides evidence for partial mediation (Diener and Emmons, 1984).

## Discussion

These investigation's aims are two-fold. First, it aims to systematically review studies of PA interventions that use PAVs as the mediating variables to evaluate and provide narrative summaries of these studies. In these 40 included studies, similar constructs (e.g., positive affect, affective attitude, PA enjoyment, vigor, activation, excitement) were grouped into PAVs to serve as mediating variables for the PA interventions. The narrative review revealed that in exploring the mediating role of the PAVs, the vast majority of studies had focused more on the role of enjoyment and less on other similar constructs (e.g., vigor, activation, excitement). Moreover, the majority of research subjects are students, limiting the diversity of subjects in such research, although it is easier for schools and universities to conduct experiments. Besides, only one study has focused on PAV's effect on male PA outcomes, and relatively few studies have accurately analyzed the mediating role of PAVs on the male PA outcomes. So far, we have not found a comparison of the mediating effects of PAVs on PAs between males and females, and perhaps this is a direction worth exploring. Finally, the study found a considerable variation in the frequency of use of each behavior change technique in included studies, with some being utilized by more than one-third of all studies, while the other five were not utilized by any included studies. A more detailed review summarizing the effects of each behavior change technique on PAV and PA has yet to be completed; furthermore, rigorous experimental testing using factorial designs to isolate and incorporate unique techniques is also necessary.

Second, it aims to statistically synthesize evidence for the mechanism of the effect of PAV on PA outcome. To achieve this, we constructed a framework that predicted that intervention would have initial effects on the proximal outcome or mediating mechanism (PAVs) and the distal outcomes (PA). The results showed a significant and moderate effect of PAV as a mediating variable for the PA intervention, suggesting that PAV plays a unique role in determining PA. It is a juxtaposition of findings: (a) intervention was positively associated with PAV; (b) PAV was positively associated with PA outcome; (c) intervention was positively associated with PA outcome. Those findings broadly supported the work of other studies in this area linking PAV with PA. For instance, according to Williams (2008), affective response to exercise is posited to influence exercise adherence via anticipated affective response to future exercise. Similarly, Lee et al. (2016) proposed a two-pronged approach to PA promotion. They posited that more likely those strategies result in more positive affective responses to exercise as well as better adherence of participants to exercise. These findings are also consistent with the principle of hedonism, which states that individuals seek to maximize enjoyment and minimize pain (Higgins, 1997). In the light of the current research findings that contemplate this principle, the primary purpose of PA promotion interventions is to facilitate enjoyment rather than physiological benefits (Nielsen et al., 2014), which seems sensible. Over the past decade, there has been an upsurge of enthusiasm for considering the role of positive emotions and affects in the prescription of PA more fully (e.g., Ekkekakis et al., 2013, 2020). An underlying message of these sources is that if individuals are not motivated by self-determined influences, such as enjoyment, then they are less likely to engage in long-term PA, no matter how often they are informed of its potential health benefits (Brand and Ekkekakis, 2018). Therefore, exercise interventions that promote self-determination (Ryan and Deci, 2000) have the potential to promote the maintenance of PA behaviors. In conjunction with previous meta-analysis reviews of affective variables or affective judgments (Nasuti and Rhodes, 2013; Rhodes et al., 2019b), and findings from previous meta-analyses of PA interventions (Conn et al., 2011), these studies support the central role played by PAVs.

## Limitations and Future Research Directions

To reduce the possibility of selection bias, we used systematic and comprehensive search techniques to locate studies, although it may not be possible to identify all substantial investigations. The decision to exclude studies published in languages other than English was considered a minor limitation. Besides, this paper focuses on subjects in non-clinical states and does not explore and calculate the mediating effects of PAVs on clinical exercise interventions. Such studies would hold particular value, if they focused on clinical populations, including diabetics, the clinically obese, and other patients recovering from surgery (Hutchinson et al., 2017). Furthermore, given that most of the subjects in the studies included in this paper were female or of mixed-gender, it is also necessary to distinguish between the role of PAVs for male and female exercise in future studies.

## Conclusion

Overall, the findings suggest that intervention can moderately increase PAV in exercisers, PAV can moderately boost PA in exercisers, intervention can slightly increase PA in exercisers, and PAV partially mediates between intervention and PA improvement. Given the summative evidence in the research literature supporting PAVs for a range of PA outcomes, it is reasonable to conclude that PAV increasement intervention has the capacity to provide considerable positive effects for exercisers to improve PA outcomes. This study has identified and highlighted that PAV can be a mediator between intervention and PA, which means that we can direct better and stronger interventions that trigger key PA change processes. Thus, it is strongly recommended that future interventions be more innovative and aim for higher fidelity with PAV as a mediator.

## Data Availability Statement

All datasets presented in this study are included in the article/Supplementary Material.

## Author Contributions

DJ and CC contributed to conception and design of the study. DJ supervised the entire process. CC organized the database, performed the statistical analysis, and wrote the manuscript. EF and AK supported CC in data extraction and data analysis phase. DJ, EF, and CC contributed to manuscript revision. All authors read and approved the submitted version.

## Conflict of Interest

The authors declare that the research was conducted in the absence of any commercial or financial relationships that could be construed as a potential conflict of interest.
