# Supplementary material for: Can Positive Affective Variables Mediate Intervention Effects on Physical Activity? A Systematic Review and Meta-Analysis
Source: Front Psychol. 2020 Nov 5;11:587757. doi: 10.3389/fpsyg.2020.587757 (PMC7674307; doi:10.3389/fpsyg.2020.587757)
Supplement: Supplementary file 1 [file Table_1.docx]

**Appendix 1**

*General Study Characteristics*

|  | Study | |  | Participants in Intervention Group | | | |  | Intervention | |  | Affective dimensions | |  | PA | |
| --- | --- | --- | --- | --- | --- | --- | --- | --- | --- | --- | --- | --- | --- | --- | --- | --- |
|  | Primary Author &  Year | Setting |  | PA Level | N | Female % | M_age_ (SD) |  | Theory | Length |  | Type | Measure |  | Type | Measure |
| 1 | Berg et al., 2020 | Internet |  | Unreported | 226 | 96.02 | 27±6.68 |  | SDT, DMP | 4 weeks |  | Positive affects | PANAS-X |  | PA engagement | PA time-consuming questionnaire |
|  |  |  |  |  |  |  |  |  |  |  |  |  |  |  |  |  |
| 2 | Faro et al., 2019 | University |  | Not meeting guideline | 34 | 100 | 27.3±4.5 |  | DMM | 4 weeks |  | Enjoyment | PACES, FS |  | RPE, HR | RPES, HRM |
|  |  |  |  |  |  |  |  |  |  |  |  |  |  |  |  |  |
| 3 | Gråstén et al., 2019 | School |  | Mixed | 661 | 52.60 | 12.12±0.33 |  | AGT, SEM | 2 years |  | Enjoyment | PEES |  | MVPA | HBSC, Accelerometer |
|  |  |  |  |  |  |  |  |  |  |  |  |  |  |  |  |  |
| 4 | Invernizzi et al., 2019 | School |  | mixed | 62 | 46.77 | 10.5±0.5 |  | CPT | 12 weeks |  | Enjoyment | PACES |  | PA | PAQ-C |
|  |  |  |  |  |  |  |  |  |  |  |  |  |  |  |  |  |
| 5 | Keeney et al., 2019 | University |  | Unreported | 36 | 15.1 | 34±13.7 |  | SDT, TCMM | 16-17 weeks |  | Enjoyment | IMI |  | PA | Pedometer |
|  |  |  |  |  |  |  |  |  |  |  |  |  |  |  |  |  |
| 6 | Robbins et al., 2019 | School & internet |  | Mixed | 1519 | 100 | 12.05±1.01 |  | HPM, SDT | 17 weeks |  | Enjoyment | PACES |  | MVPA | Accelerometer |
|  |  |  |  |  |  |  |  |  |  |  |  |  |  |  |  |  |
| 7 | Rodríguez et al., 2019 | School |  | Mixed | 131 | 51.91 | 8.66±1.77 |  | TGM | 8 weeks |  | Affective valence | FS |  | PA | Pedometer |
|  |  |  |  |  |  |  |  |  |  |  |  |  |  |  |  |  |
| 8 | Vazou et al., 2019 | School |  | Mixed | 148 | 52% | 10.39±0.98 |  | ART | 30 minutes |  | Enjoyment | S-PACES |  | PA | Accelerometer |
|  |  |  |  |  |  |  |  |  |  |  |  |  |  |  |  |  |
| 9 | Vitali et al., 2019 | School |  | Mixed | 80 | 48.75 | 10.45±0.23 |  | Null | 4 years |  | Enjoyment | PACES |  | PA | CLASS |
|  |  |  |  |  |  |  |  |  |  |  |  |  |  |  |  |  |
| 10 | Andruschko et al., 2018 | School |  | Not meeting guideline | 20 | 100 | 13.2±0.9 |  | SCT | 6 months |  | Enjoyment | Likert scale |  | PA, MVPA | Accelerometer |
|  |  |  |  |  |  |  |  |  |  |  |  |  |  |  |  |  |
| 11 | Hutchinson et al., 2018 | Lab |  | Meeting Guideline | 17 | 47.1 | 28.1±9.9 |  | HT, DMM | 48 hours |  | Remembered pleasure | VAS |  | HR | HRM |
|  |  |  |  |  |  |  |  |  |  |  |  |  |  |  |  |  |
| 12 | Miragall et al., 2018 | Internet & university |  | Not meeting guideline | 76 | 85.5 | 22.18±3.71 |  | TTM | 3 weeks |  | Enjoyment | PACES |  | PA | Pedometer |
|  |  |  |  |  |  |  |  |  |  |  |  |  |  |  |  |  |
| 13 | Rhodes et al., 2019 | Family |  | Not meeting guideline | 73 | Unreported | 11.5±1.3 |  | TPB, SDT | 13 weeks |  | Affective attitude | SD differentia |  | Equipment usage | Exercise log |
|  |  |  |  |  |  |  |  |  |  |  |  |  |  |  |  |  |
| 14 | Billing, 2017 | Telephone |  | Not meeting guideline | 40 | 90 | 39±12 |  | DMM, HT, SCT | 12 weeks |  | Enjoyment | PACES |  | MVPA | Accelerometer, 7DPAR |
|  |  |  |  |  |  |  |  |  |  |  |  |  |  |  |  |  |
| 15 | Niedermeier et al., 2017 | Outdoor, lab |  | Mixed | 42 | 48 | 32.00±11.90 |  | DMM, CM | 170 minutes |  | Mood state | MSS |  | HR, RPE | RPES |
|  |  |  |  |  |  |  |  |  |  |  |  |  |  |  |  |  |
| 16 | Noradechanunt et al., 2017 | Community |  | Not meeting guideline | 39 | 74.36 | 66.6±6.7 |  | Null | 12 weeks |  | Enjoyment | PACES |  | PA | PASE |
|  |  |  |  |  |  |  |  |  |  |  |  |  |  |  |  |  |
| 17 | Jekauc, 2015 | Community |  | Unreported | 41 | 87.8 | 46.12 |  | SDT | 8 weeks |  | Enjoyment | PACES |  | Exercise Adherence | Attendance lists |
|  |  |  |  |  |  |  |  |  |  |  |  |  |  |  |  |  |
| 18 | Kraft et al., 2015 | University |  | Mixed | 20 | 50 | 22.06±3.6 |  | Null | 15minutes*3 |  | Enjoyment | VAS |  | HR, RPE, MET | HRM, RPES, Accelerometer |
|  |  |  |  |  |  |  |  |  |  |  |  |  |  |  |  |  |
| 19 | Wang et al., 2015 | School |  | Unreported | 62 | 50 | 22.3±1.51 |  | SDT, SNST | 8 weeks |  | Enjoyment | IMI |  | PA | IPAQ |
|  |  |  |  |  |  |  |  |  |  |  |  |  |  |  |  |  |
| 20 | Mark et al., 2013 | Family |  | Not meeting guideline | 30 families | 50.84 | 36.83±6.30 |  | TPB | 6 weeks |  | affective attitude | Likert scale |  | leisure-time PA | GLTEQ |
|  |  |  |  |  |  |  |  |  |  |  |  |  |  |  |  |  |
| 21 | Bergh et al., 2012 | School |  | Unreported | 215 | 60 | 11.±6.3 |  | Null | 20 months |  | Enjoyment | SD |  | PA | Accelerometer |
|  |  |  |  |  |  |  |  |  |  |  |  |  |  |  |  |  |
| 22 | Fitzsimons et al., 2012 | Community |  | Not meeting guideline | 79 | 88.73 | 49±9 |  | TTM | 48 weeks |  | Affect | PANAS |  | PA | Pedometer |
|  |  |  |  |  |  |  |  |  |  |  |  |  |  |  |  |  |
| 23 | Conner et al., 2011 | University |  | Not meeting guideline | 316 | 64.24 | 22 |  | TPB | 3 weeks |  | Affective attitude | SD |  | PA | GLTEQ |
|  |  |  |  |  |  |  |  |  |  |  |  |  |  |  |  |  |
| 24 | Schneider et al., 2011 | School |  | Not meeting guideline | 122 | 100 | 15.04±0.78 |  | HT, SDT | 9 months |  | Enjoyment | PACES |  | PA | 3DPAR |
|  |  |  |  |  |  |  |  |  |  |  |  |  |  |  |  |  |
| 25 | Louise et al., 2010 | School |  | Unreported | 221 | 59.28 | 13.29±0.99 |  | SMT | 16 weeks |  | Enjoyment | PACES |  | LTPA | 7DPAR |
|  |  |  |  |  |  |  |  |  |  |  |  |  |  |  |  |  |
| 26 | Sirriyeh et al., 2010 | School |  | Unreported | 31 | 70 | 17.3±0.68 |  | TPB | 14 days |  | enjoyable | Unreported |  | PA | IPAQ |
|  |  |  |  |  |  |  |  |  |  |  |  |  |  |  |  |  |
| 27 | Focht, 2009 | Lab & Outdoor |  | Meeting guideline | 35 | 100 | 22.14±1.73 |  | TPB | 10 minutes |  | Enjoyment | SES |  | PA, HR | LTEQ, HRM |
|  |  |  |  |  |  |  |  |  |  |  |  |  |  |  |  |  |
| 28 | Rhodes, Warburton, & Bredin, 2009 | University |  | Meeting guideline | 29 | 0 | 22.7±4.0 |  | TPB | 6 weeks |  | Affective attitude | SD |  | Adherence to exercise | Attendance list |
|  |  |  |  |  |  |  |  |  |  |  |  |  |  |  |  |  |
| 29 | Annesi et al., 2008 | Community |  | Unreported | 269 | 59 | 10.6±1.1 |  | SET, SCT | 1 year |  | Vigor | POMS |  | Voluntary Physical Activity | SSMVPA |
|  |  |  |  |  |  |  |  |  |  |  |  |  |  |  |  |  |
| 30 | Baker et al., 2008 | Community |  | Not meeting guideline | 79 | 79.75 | 49.2±8.9 |  | TTM | 12 weeks |  | Affect | PANAS |  | PA | Pedometer |
|  |  |  |  |  |  |  |  |  |  |  |  |  |  |  |  |  |
| 31 | Edmunds et al., 2008 | University |  | Mixed | 56 | 100 | 21.32±5.56 |  | SDT | 10 weeks |  | Affect | PANAS |  | Exercise Behavior | Attendance list |
|  |  |  |  |  |  |  |  |  |  |  |  |  |  |  |  |  |
| 32 | Duntion et al., 2007 | School |  | Not meeting guideline | 79 | Unreported | 10^th^ or 11^th^ students |  | SCT, SEM | 3 years |  | Enjoyment | PACES |  | PA | 3DPAR |
|  |  |  |  |  |  |  |  |  |  |  |  |  |  |  |  |  |
| 33 | Rose et al., 2007 | Lab |  | Not meeting guideline | 19 | 100 | 39.37±10.29 |  | DMM, SCT, SET | 20 minutes |  | Affective valence | FS |  | RPE | RPES |
|  |  |  |  |  |  |  |  |  |  |  |  |  |  |  |  |  |
| 34 | Focht et al., 2007 | Lab |  | Not meeting guideline | 18 | 55.56 | 24.10±3.40 |  | SCT | 8 weeks |  | Exercise-induced feelings | EFI |  | RPE | RPES |
|  |  |  |  |  |  |  |  |  |  |  |  |  |  |  |  |  |
| 35 | Robbins et al., 2006 | School |  | Not meeting guideline | 77 | 100 | 12.13±0.91 |  | HPM, TTM, SCT | 12 weeks |  | Enjoyment | PACES |  | PA | CAAL |
|  |  |  |  |  |  |  |  |  |  |  |  |  |  |  |  |  |
| 36 | Dishman et al., 2005 | School |  | Unreported | 1049 | 100 | 13.6±0.6 |  |  | 1 year |  | Enjoyment | PACES |  | PA | 3DPAR |
|  |  |  |  |  |  |  |  |  |  |  |  |  |  |  |  |  |
| 37 | Jamner et al., 2004 | School |  | Not meeting guideline | 58 | 100 | 14.94±0.79 |  | Null | 4 months |  | Enjoyment | PACES |  | PA | 2DPAR, SUPAS |
|  |  |  |  |  |  |  |  |  |  |  |  |  |  |  |  |  |
| 38 | Digelidis et al., 2003 | School |  | Unreported | 782 | 52.17 | 12.05±0.73 |  | TPB, GPT, TARGETM | 1 year |  | Enjoyment | IMI |  | Exercise behavior | EFS |
|  |  |  |  |  |  |  |  |  |  |  |  |  |  |  |  |  |
| 39 | McAuley et al., 2003 | Gymnasium |  | Not meeting guideline | 174 | 71.84 | 65.5 |  | SCT | 6 months |  | Exercise affect | FS |  | Exercise frequency | Exercise log |
|  |  |  |  |  |  |  |  |  |  |  |  |  |  |  |  |  |
| 40 | Nichols et al., 2000 | Worksite |  | Not meeting guideline | 160 | 78.13 | 42.0±9.7 |  | SCT, TTM | 33 months |  | Enjoyment | PACES |  | PA | 7DPAR |

*Note.* PAQ-C = The physical activity questionnaire for children; CLASS = The children’s leisure activities study survey; RPES = Ratings of perceived exertion scale; 7DPAR = 7-day physical activity recall; 3DPAR = 3-day physical Activity Recall; 2DPAR = 2-day physical activity recall; HRM = Heart rate monitor; HBSC = The health behavior in School-aged children research protocol; PASE = The physical activity scale for the elderly; IPAQ = The short-form of the international physical activity questionnaire; GLTEQ = A modified Godin leisure-time exercise questionnaire; LTEQ = Leisure-time exercise questionnaire; SSMVPA = A single-item scale to assess the moderate to vigorous physical activity over the previous week; CAAL = the child and adolescent activity log; SUPAS = the Stanford usual physical activity scale; EFS = 6-point exercise frequency scale; PACES = The physical activity enjoyment scale; S-PACES = Shorted physical activity enjoyment scale for children; FS = The feeling scale; PEES = The PE enjoyment scale; DMP = Dualistic Model of Passion; VAS = Visual analog scale; MSS = A mood survey scale; IMI = The intrinsic motivation inventory; EFI = The Exercise-induced Feeling Inventory; PANAS = The positive and negative affect schedule; PANAS-X = The positive and negative affect schedule-expanded form; TCMM = The trans-contextual model of motivation;SES = Single-item enjoyment scale; POMS = The tension and vigor scales of the profile of mood states-short Form; CPT = Challenge point theory; DMM = The dual-mode model; HPM = the health promotion model; SMT = Self-management theory; PMT = Protection motivation theory; SDT = Self-determination Theory; TGM = Tactical games model; ART = Affective reflective theory; AGT = Achievement goal theory; SEM = Social ecological model; SCT = Social cognitive theory; TTM = The transtheoretical model; TPB = Theory of planned behavior; HT = The hedonic theory; CM = The circumplex model; SNST = Social network site theory; SMT = Social marketing theory; SET = Self-efficacy theory; GPT = Goal perspectives theory; TARGETM = The TARGET model

**Appendix 2**

*Intervention techniques included in each study*

|  | Study | Intervention techniques |
| --- | --- | --- |
| 1 | Berg et al., 2020 | 16, 34, 37 |
| 2 | Faro et al., 2019 | 16, 20, 21, 22 |
| 3 | Gråstén et al., 2019 | 7, 8, 16, 19, 20, 21, 22, 24, 26, 29, 36, 39 |
| 4 | Invernizzi et al., 2019 | 1, 5, 7, 8, 9, 16, 19, 20, 21, 22, 28, 29, 36 |
| 5 | Keeney et al., 2019 | 6, 11, 17, 18, 19, 28, 29 |
| 6 | Robbins et al., 2019 | 2, 8, 16, 19, 29, 36, 37 |
| 7 | Rodríguez et al., 2019 | 10, 20, 21, 22, 26 |
| 8 | Vazou et al., 2019 | 3, 7, 13, 16, 19, 20, 21, 22 |
| 9 | Vitali et al., 2019 | 1, 5, 10, 20, 21, 22, 23, 29 |
| 10 | Andruschko et al., 2018 | 5, 7, 8, 16, 19, 20, 21, 22, 29, 36, 37, 38 |
| 11 | Hutchinson et al., 2018 | 10, 16, 20, 21, 24, 36 |
| 12 | Miragall et al., 2018 | 1, 5, 6, 16, 19, 36 |
| 13 | Rhodes et al., 2019 | 5, 7, 8, 10, 16, 20, 21, 24,29, 34 |
| 14 | Billing, 2017 | 5, 6, 7, 10, 12, 16, 23, 27, 29, 34, 36 |
| 15 | Niedermeier et al., 2017 | 5, 9, 20, 21, 24, 29 |
| 16 | Noradechanuntet al., 2017 | 7, 20, 21, 22, 27 |
| 17 | Jekauc, 2015 | 7,10,11,18,19,20,21, 22, 28,36 |
| 18 | Kraft et al., 2015 | 7,10,11,18,19,20,21, 22, 28,36 |
| 19 | Wang et al., 2015 | 1, 3, 4, 7, 20, 21, 22, 23, 25, 28, 29, 36 |
| 20 | Mark et al., 2013 | 13, 16, 20, 21, 24, 34 |
| 21 | Bergh et al., 2012 | 7, 12, 20, 21, 24, 29 |
| 22 | Fitzsimons et al., 2012 | 7, 8, 9, 10, 16, 17, 19, 21, 22, 29, 35, 36, 37 |
| 23 | Conner et al., 2011 | 1, 34, 36 |
| 24 | Schneider et al., 2011 | 1, 3, 5, 7, 8, 16, 19, 20, 21, 22, 29, 36 |
| 25 | Louise et al., 2010 | 1, 8, 10, 19, 20, 21, 22, 24, 29, 36, 39 |
| 26 | Sirriyeh et al., 2010 | 1, 36 |
| 27 | Focht, 2009 | 20, 24 |
| 28 | Rhodes, Warburton, & Bredin, 2009 | 7, 16, 20, 21, 24, 34 |
| 29 | Annesi et al., 2008 | 1, 4, 5, 6, 7, 10, 11, 19, 20, 21, 22, 26, 29, 33 |
| 30 | Baker et al., 2008 | 7, 8, 9, 10, 16, 17, 19, 21, 22, 29, 35, 36, 37 |
| 31 | Edmunds et al., 2008 | 4, 6, 7, 10, 11, 19, 20, 21, 22, 26, 29, 33 |
| 32 | Dunton et al., 2007 | 7, 8, 14, 20, 21, 22, 29, 36, 38 |
| 33 | Rose et al., 2007 | 5, 20, 21, 36 |
| 34 | Focht et al., 2007 | 7, 20, 21 |
| 35 | Robbins et al., 2006 | 1, 2, 3, 4, 7, 8, 19, 20, 21, 22, 24, 29, 36 |
| 36 | Dishman et al., 2005 | 1, 7, 20, 21, 22, 24, 29 |
| 37 | Jamner et al., 2004 | 1, 3, 5, 7, 8, 16, 19, 20, 21, 22, 29, 36 |
| 38 | Digelidis et al., 2003 | 1, 5, 6, 7, 9, 17, 19, 20, 21, 22, 29, 33, 34, 36, 38, 39 |
| 39 | McAuley et al., 2003 | 7, 9, 20, 21, 22 |
| 40 | Nichols et al., 2000 | 1, 3, 5, 7, 8, 16, 18, 20, 21, 22, 23, 24, 26, 29, 33, 38, 39 |

*Note.* The symbolic coding corresponds to the following behavior change strategies: 1_Provide information on consequences of behavior in general; 2_Provide information on consequences of behavior to individual; 3_Provide information about others’ approval; 4_Provide normative information about others’ behavior; 5_Goal setting (behavior); 6_Goal setting (outcome); 7_Action planning; 8_Barrier identification/problem solving; 9_Set graded tasks; 10_Prompt review of behavioral goals; 11_Prompt review of outcome goals; 12_Provide rewards contingent on effort or progress towards behavior; 13_Provide rewards contingent on successful behavior; 14_Shaping; 15_Prompt generalization of a target behavior; 16_Prompt self-monitoring of behavior; 17_Prompt self-monitoring of behavioral outcome; 18_Prompting focus on past success; 19_Provide feedback on performance; 20_Provide instruction on when and where to perform the behavior; 21_Provide instruction on how to perform the behavior; 22_Model/demonstrate the behavior; 23_Teach to use prompts/cues; 24_Environmental restructuring; 25_Agree behavioral contract; 26_Prompt practice; 27_Use of follow-up prompts; 28_Facilitate social comparison; 29_Plan social support/social change; 30_Prompt identification as a role model/position advocate; 31_Prompt anticipated regret; 32_Fear arousal; 33_Prompt self-talk; 34_Prompt use of imagery; 35_Relapse prevention/coping planning; 36_Stress management/ emotional training; 37_Motivational interviewing; 38_Time management; 39_General communication skills training; 40_Stimulate anticipation of future rewards

**Appendix 3**

*References for Articles included in meta-analytic mediation analyses*

1. Berg, S., Forest, J., & Stenseng, F. (2020). When Passion Does Not Change, but Emotions Do: Testing a Social Media Intervention Related to Exercise Activity Engagement. *Frontiers in Psychology*, *11*, 71.
2. Faro, J., Wright, J. A., Hayman, L. L., Hastie, M., Gona, P. N., & Whiteley, J. A. (2019). Functional resistance training and affective response in female college-age students. *Medicine and science in sports and exercise*, *51*(6), 1186.
3. Gråstén, A., & Yli‐Piipari, S. (2019). The Patterns of Moderate to Vigorous Physical Activity and Physical Education Enjoyment Through a 2‐Year School‐Based Program. *Journal of School Health*, *89*(2), 88-98.
4. Invernizzi, P. L., Crotti, M., Bosio, A., Cavaggioni, L., Alberti, G., & Scurati, R. (2019). Multi-teaching styles approach and active reflection: Effectiveness in improving fitness level, motor competence, enjoyment, amount of physical activity, and effects on the perception of physical education lessons in primary school children. *Sustainability*, *11*(2), 405.
5. Keeney, J., Schneider, K. L., & Moller, A. C. (2019). Lessons learned during formative phase development of an asynchronous, active video game intervention: Making sedentary fantasy sports active. *Psychology of Sport and Exercise*, *41*, 200-210.
6. Robbins, L. B., Wen, F., & Ling, J. (2019). Mediators of physical activity behavior change in the “Girls on the Move” intervention. *Nursing research*, *68*(4), 257-266.
7. Rodríguez-Negro, J., & Yanci, J. (2020). Which instructional models influence more on perceived exertion, affective valence, physical activity level, and class time in physical education?. *Educational Psychology*, *40*(5), 608-621.
8. Vazou, S., Mischo, A., Ladwig, M. A., Ekkekakis, P., & Welk, G. (2019). Psychologically informed physical fitness practice in schools: A field experiment. *Psychology of Sport and Exercise*, *40*, 143-151.
9. Vitali, F., Robazza, C., Bortoli, L., Bertinato, L., Schena, F., & Lanza, M. (2019). Enhancing fitness, enjoyment, and physical self-efficacy in primary school children: a DEDIPAC naturalistic study. *PeerJ*, *7*, e6436.
10. Andruschko, J., Okely, A. D., & Pearson, P. (2018). A school-based physical activity and motor development program for low-fit adolescent females: the Sport4Fun pilot randomized controlled trial. *Journal of Motor Learning and Development*, *6*(2), 345-356.
11. Hutchinson, J. C., Jones, L., Vitti, S. N., Moore, A., Dalton, P. C., & O'Neil, B. J. (2018). The influence of self-selected music on affect-regulated exercise intensity and remembered pleasure during treadmill running. *Sport, Exercise, and Performance Psychology*, *7*(1), 80.
12. Miragall, M., Domínguez-Rodríguez, A., Navarro, J., Cebolla, A., & Baños, R. M. (2018). Increasing physical activity through an internet-based motivational intervention supported by pedometers in a sample of sedentary students: A randomised controlled trial. *Psychology & health*, *33*(4), 465-482.
13. Rhodes, R. E., Beauchamp, M. R., Blanchard, C. M., Bredin, S. S., Warburton, D. E., & Maddison, R. (2019). Predictors of stationary cycling exergame use among inactive children in the family home. *Psychology of Sport and Exercise*, *41*, 181-190.
14. Billing, L. (2017). The Efficacy of Affective Behavioral Strategies for Increasing Physical Activity: Implications for Harnessing the Dual-Mode Model.
15. Niedermeier, M., Einwanger, J., Hartl, A., & Kopp, M. (2017). Affective responses in mountain hiking—A randomized crossover trial focusing on differences between indoor and outdoor activity. *PLoS One*, *12*(5), e0177719.
16. Noradechanunt, C., Worsley, A., & Groeller, H. (2017). Thai Yoga improves physical function and well-being in older adults: A randomised controlled trial. *Journal of science and medicine in sport*, *20*(5), 494-501.
17. Jekauc, D. (2015). Enjoyment during exercise mediates the effects of an intervention on exercise adherence. *Psychology*, *6*(01), 48.
18. Kraft, J. A., Russell, W. D., Clark, N., Helm, J., & Jackson, A. (2015). Influence of experience level on physical activity during interactive video gaming. *Journal of Physical Activity and Health*, *12*(6), 794-800.
19. Wang, J. C., Leng, H. K., & Kee, Y. H. (2015). Use of Facebook in physical activity intervention programme: Test of self-determination theory.
20. Mark, R. S., & Rhodes, R. E. (2013). Testing the effectiveness of exercise videogame bikes among families in the home-setting: a pilot study. *Journal of Physical Activity and Health*, *10*(2), 211-221.
21. Bergh, I. H., van Stralen, M. M., Grydeland, M., Bjelland, M., Lien, N., Andersen, L. F., ... & Ommundsen, Y. (2012). Exploring mediators of accelerometer assessed physical activity in young adolescents in the health in adolescents study–a group randomized controlled trial. *BMC Public Health*, *12*(1), 814.
22. Fitzsimons, C. F., Baker, G., Gray, S. R., Nimmo, M. A., & Mutrie, N. (2012). Does physical activity counselling enhance the effects of a pedometer-based intervention over the long-term: 12-month findings from the Walking for Wellbeing in the west study. *BMC public health*, *12*(1), 1-12.
23. Conner, M., Rhodes, R. E., Morris, B., McEachan, R., & Lawton, R. (2011). Changing exercise through targeting affective or cognitive attitudes. *Psychology and Health*, *26*(2), 133-149.
24. Schneider, M., & Cooper, D. M. (2011). Enjoyment of exercise moderates the impact of a school-based physical activity intervention. *International Journal of Behavioral Nutrition and Physical Activity*, *8*(1), 1-8.
25. Louise Bush, P., Laberge, S., & Laforest, S. (2010). Physical activity promotion among underserved adolescents:“make it fun, easy, and popular”. *Health Promotion Practice*, *11*(3_suppl), 79S-87S.
26. Sirriyeh, R., Lawton, R., & Ward, J. (2010). Physical activity and adolescents: an exploratory randomized controlled trial investigating the influence of affective and instrumental text messages. *British journal of health psychology*, *15*(4), 825-840.
27. Focht, B. C. (2009). Brief walks in outdoor and laboratory environments: effects on affective responses, enjoyment, and intentions to walk for exercise. *Research quarterly for exercise and sport*, *80*(3), 611-620.
28. Rhodes, R. E., Warburton, D. E., & Bredin, S. S. (2009). Predicting the effect of interactive video bikes on exercise adherence: An efficacy trial. *Psychology, health & medicine*, *14*(6), 631-640.
29. Annesi, J. J., Faigenbaum, A. D., Westcott, W. L., & Smith, A. E. (2008). Relations of self-appraisal and mood changes with voluntary physical activity changes in African American preadolescents in an after-school care intervention. *Journal of sports science & medicine*, *7*(2), 260.
30. Baker, G., Gray, S. R., Wright, A., Fitzsimons, C., Nimmo, M., Lowry, R., ... & Scottish Physical Activity Research Collaboration. (2008). The effect of a pedometer-based community walking intervention" Walking for Wellbeing in the West" on physical activity levels and health outcomes: a 12-week randomized controlled trial. *International Journal of Behavioral Nutrition and Physical Activity*, *5*(1), 44.
31. Edmunds, J., Ntoumanis, N., & Duda, J. L. (2008). Testing a self‐determination theory‐based teaching style intervention in the exercise domain. *European journal of social psychology*, *38*(2), 375-388.
32. Dunton, G. F., Schneider, M., & Cooper, D. M. (2007). An investigation of psychosocial factors related to changes in physical activity and fitness among female adolescents. *Psychology and Health*, *22*(8), 929-944.
33. Rose, E. A., & Parfitt, G. (2007). A quantitative analysis and qualitative explanation of the individual differences in affective responses to prescribed and self-selected exercise intensities. *Journal of Sport and Exercise Psychology*, *29*(3), 281-309.
34. Focht, B. C., Knapp, D. J., Gavin, T. P., Raedeke, T. D., & Hickner, R. C. (2007). Affective and self-efficacy responses to acute aerobic exercise in sedentary older and younger adults. *Journal of Aging and Physical Activity*, *15*(2), 123-138.
35. Robbins, L. B., Gretebeck, K. A., Kazanis, A. S., & Pender, N. J. (2006). Girls on the move program to increase physical activity participation. *Nursing research*, *55*(3), 206-216.
36. Dishman, R. K., Motl, R. W., Saunders, R., Felton, G., Ward, D. S., Dowda, M., & Pate, R. R. (2005). Enjoyment mediates effects of a school-based physical-activity intervention. *Medicine and science in sports and exercise*, *37*(3), 478-487.
37. Jamner, M. S., Spruijt-Metz, D., Bassin, S., & Cooper, D. M. (2004). A controlled evaluation of a school-based intervention to promote physical activity among sedentary adolescent females: project FAB. *Journal of adolescent health*, *34*(4), 279-289.
38. Digelidis, N., Papaioannou, A., Laparidis, K., & Christodoulidis, T. (2003). A one-year intervention in 7th grade physical education classes aiming to change motivational climate and attitudes towards exercise. *Psychology of Sport and exercise*, *4*(3), 195-210.
39. McAuley, E., Jerome, G. J., Marquez, D. X., Elavsky, S., & Blissmer, B. (2003). Exercise self-efficacy in older adults: social, affective, and behavioral influences. *Annals of Behavioral Medicine*, *25*(1), 1.
40. Nichols, J. F., Wellman, E., Caparosa, S., Sallis, J. F., Calfas, K. J., & Rowe, R. (2000). Impact of a worksite behavioral skills intervention. *American Journal of Health Promotion*, *14*(4), 218-221.
